# Update on epidemiology of canine babesiosis in Southern France

**DOI:** 10.1186/s12917-015-0525-3

**Published:** 2015-08-25

**Authors:** Magalie René-Martellet, Claire Valiente Moro, Jeanne Chêne, Gilles Bourdoiseau, Luc Chabanne, Patrick Mavingui

**Affiliations:** Université de Lyon, VetAgro Sup, Jeune équipe Hémopathogènes vectorisés, Marcy l’Etoile, France; Université de Lyon, Ecologie microbienne, UMR CNRS 5557, USC INRA 1364, VetAgro Sup, Université Lyon 1, Villeurbanne, France; Université de La Réunion, Unité Mixte de Recherche Processus Infectieux en Milieu Insulaire Tropical (UMR PIMIT), INSERM 1187, CNRS 9192, IRD 249, Plateforme de Recherche CYROI, Saint-Denis, 97490 Ste Clotilde, La Réunion France; INRA, UR 0346 Epidémiologie Animale, 63122 Saint-Genès-Champanelle, France

**Keywords:** Canine babesiosis, *Rhipicephalus sanguineus*, *Dermacentor reticulatus*, *Babesia vogeli*, *Babesia canis*, Molecular characterization, Epidemiology

## Abstract

**Background:**

Canine babesiosis is an emerging or re-emerging disease caused by *Babesia* and *Theileria* protozoans, also called piroplasms, transmitted by Ixodid ticks. In Europe, four etiological agents have been identified to date, namely *Babesia canis, B. vogeli, B. gibsoni* and *Theileria annae.* France has a high prevalence of canine babesiosis and two tick species, *Dermacentor reticulatus* and *Rhipicephalus sanguineus*, are supposed to transmit *B. canis* and *B. vogeli* respectively. In southern France, where dog infections with *B. vogeli* were recently confirmed, no comprehensive study was performed to date on piroplasm species infecting dogs. Thus, a large scale survey involving veterinary clinics, kennels and tick collection from the environment was conducted from 2010 to 2012 in this area.

**Results:**

From 2010 to 2012, 140 dog blood samples and 667 ticks were collected. All blood and a subset of ticks were screened for the presence of piroplasms by PCR amplification of 18S rDNA. *B. vogeli*, *B. canis* and *T. annae* were detected in 13.6, 12.9 and 0.7 % dogs respectively. *B. vogeli* and *B. canis* were detected in 10.5 % and in 1.6 % *R. sanguineus* ticks including 1.3 % co-infections. *B. canis* was the only species detected in *D. reticulatus* ticks (9.7 %). *B. canis* infections were only recorded in the southwest of France whereas *B. vogeli* was mainly found in the southeast. Finally, a significantly higher prevalence of *B. vogeli* infection was found in Gard compared to Corsica and Drôme regions, both in dogs (*p* < 0.002) and *R. sanguineus* ticks (*p* < 0.02) although *R. sanguineus* was the main ticks species removed from dogs in those three areas.

**Conclusions:**

The survey confirmed the circulation of both *B. canis* and *B. vogeli* in dogs in southern France with differences in distribution probably linked to the distribution of their respective vectors. It also showed differences in prevalence of *B. vogeli* infection in areas similar in terms of risk of dogs infestation with *R. sanguineus*. Further studies focusing on genetic and microbiota of *R. sanguineus* ticks should be conducted to explore other biological interactions that may explain the differences observed.

## Background

Babesiosis (or piroplasmosis) is an emerging or re-emerging tick-borne disease caused by intraerythrocytic protozoa of the genera *Babesia* and *Theileria*, also known as piroplasms [1]. In dogs, infection by these parasites usually induces a syndrome characterized by hyperthermia and anaemia that can be fatal when complicated [1]. The severity of the disease depends on various factors including the *Babesia*/*Theileria* species involved and the age and immune status of the host [1]. In the last few decades, thanks to molecular biology, new piroplasma species were evidenced bringing to at least seven the number of species able to infect dogs in the world [1–3]. In Europe, four of these species have been identified to date, namely *B. canis, B. vogeli, B. gibsoni* and *B. microti-*like also known as *Theileria annae*, *Babesia* sp. “Spanish dog” or *Babesia vulpes* sp. nov [3, 4]. *B. canis* is the most widely distributed species coinciding with the distribution of its known vector *Dermacentor reticulatus* [5, 6]*. B. vogeli* is generally found around the Mediterranean basin where *R. sanguineus* is the predominant tick species [3]. Infections by *B. gibsoni* and *B. microti-*like seem to remain sporadic and their vectors are currently unknown. In addition, DNA of *Theileria equi*, *Babesia caballi* and *Babesia rossi* have been very exceptionally detected in dog blood samples in Europe however the epidemiological significance of these observations is unclear [7, 8].

France has a high prevalence of canine babesiosis overall but incidence rates vary considerably according to the seasons and locality [9–11]. Although, the disease occurs almost in the whole country, more cases are generally reported in the southwest, including west of the Mediterranean basin, while they are more scarce in Corsica and east of the Mediterranean basin [12–14]. Both vectors, *D. reticulatus* and *R. Sanguineus,* are supposed to transmit *Babesia canis* and *Babesia vogeli* respectively in the country but knowledge on their distribution, pathogens they transmit as well as climatic conditions favorable to transmission is still lacking, especially in southern France where reports are scarce or interested small foci [5, 8, 9, 15]. Moreover, the taxonomic status of *R. sanguineus* remain controversial and it is now clearly established that several species are gathered into the so called *R. sanguineus* group [16]. In particular, it was shown that ticks of the *R. sanguineus* group can be divided into two main lineages: ticks of “temperate” and “tropical” areas [17–19]. The impact of those genetic variations of ticks among the *R. sanguineus* group on *B. vogeli* transmission has not been evaluated to date. In the continuity of our previous reports [11, 15] a large scale survey was conducted in the French Mediterranean basin in order to (i) enhance knowledge on prevalence of *Babesia vogeli* and other piroplasm infections in dogs (ii) try to provide key elements of response to the inhomogeneous distribution of canine babesiosis cases and (ii) appraise the importance of each vector, *R. sanguineus* and *D. reticulatus*, in the transmission of the disease in this region.

From 2010 to 2012, dog blood samples and ticks were collected from veterinary practices, kennels and the environment in southern France and tested for the presence of piroplasms using molecular tools. The study confirmed the circulation of *Babesia canis* and *B. vogeli* in dogs with differences in prevalence of infections between regions. It also supports the role of both *R. sanguineus* and *D. reticulatus* ticks in the transmission of the disease in this area.

## Methods

### Sampling

**Fig. 1:**
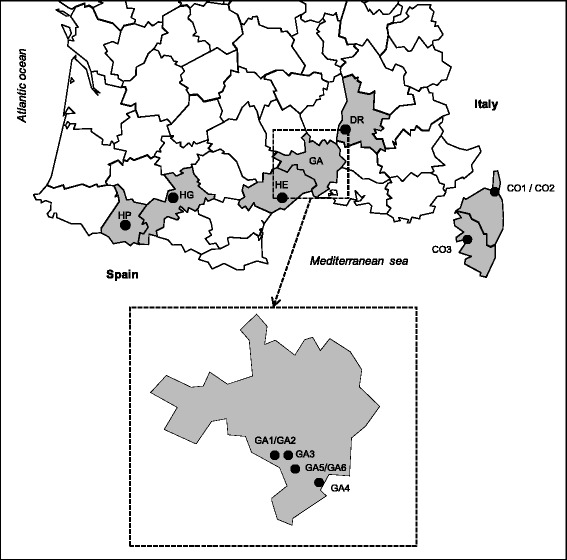
Map of sampling sites in southern France. The figure depicts southern France and its administrative divisions called “*départements*”. *Départements* where ticks and/or blood sampling where performed are indicated in grey. The survey was conducted at 13 locations indicated by black dots corresponding to veterinary practices, kennels or the environment as detailed in Table 1. Sites codes are as follows: CO, Corsica; DR, Drôme; HE, Hérault; HG, Haute-Garonne; HP, Hautes-Pyrénées; GA, Gard. We thank J.F. Bradu for providing the basemap of French *départements*

The survey was conducted at 13 locations in southern France (Fig. 1; Table 1) and involved veterinary clinics, kennels and tick collection from the environment. Study sites were selected in order to cover regions with different ecological characteristics. To enhance knowledge on prevalence of infection with *Babesia vogeli* and potential risk of babesiosis transmission to dog by *R. sanguineus* ticks, six of the sampling locations were situated in the Gard department, an area where were recently confirmed several cases of infections of dogs by this pathogen [15]. For this specific area, meteorological data (mean monthly maximum and minimum temperatures and rainfall) were obtained from the French national meteorological service (http://france.meteofrance.com/, Nîmes-Courbessac station).

**Table 1:** Sampling locations and methods

Site code	Location	Geographical coordinates	Characteristics	Collected samples	Tick sampling method
DR	La Bégude de Mazenc, Drôme	44°32′N 4°56′E	Veterinary clinic	Dog blood samples; Ticks	on dogs
HE	Béziers, Hérault	43°20′N 3°12′E	Veterinary clinic	Dog blood samples	NA
HG	Aurignac, Haute-Garonne	43°13′N 0°53′E	Veterinary clinic	Dog blood samples; Ticks	on dogs
HP	Trie sur Baïse, Hautes-Pyrénées	43°19′N 0°22′E	Veterinary clinic	Dog blood samples; Ticks	on dogs
GA1	Sommières, Gard	43°47′N 4°05′E	Veterinary clinic	Dog blood samples; Ticks	on dogs
GA2	Sommières, Gard	43°47′N 4°05′E	Along a river with reeds, in town	Ticks	flagging
GA3	Calvisson, Gard	43°47′N 4°11′E	Veterinary clinic	Dog blood samples; Ticks	on dogs
GA4	Saint-Gilles, Gard	43°40′N 4°26′E	Veterinary clinic	Dog blood samples; Ticks	on dogs
GA5	Aigues-Vives, Gard	43°42′N 4°13′E	Kennel	Dog blood samples; Ticks	on dogs; visual handling; flagging
GA6	Aigues-Vives, Gard	43°42′N 4°13′E	Along a creek, near kennel	Ticks	flagging
CO1	Bastia, Corsica	42°41′N 9°27′E	Veterinary clinic	Dog blood samples; Ticks	on dogs
CO2	Bastia, Corsica	42°41′N 9°27′E	Kennel	Dog blood samples	NA
CO3	Ajaccio, Corsica	41°55′N 8°44′E	Kennel	Dog blood samples; Ticks	on dogs

*NA* not applicable as no ticks were collected in these areas

Dogs were included in the survey in case of clinical suspicion of canine babesiosis and/or infestation with ticks. In kennels, dogs housed for more than 3 years were also selected. For all dogs included, a questionnaire was completed in order to obtain background information on pets in particular the usual place of residence, the history of any recent travel, the vaccination status regarding babesiosis and informed consent of owners. The analyses were realized in a context of diagnosis in dogs infested by ticks or suspect of canine babesiosis, thus no approval by an ethics committee was required. Finally, only dogs living around the sampling area without known travel history were kept for subsequent analyses. For each dog, blood was sampled from the cephalic or the jugular veins and stored in collection tubes with EDTA (anticoagulant) at 5 °C ± 1 °C until DNA extraction within 5 days of collection. A blood smear was also made extemporaneously with fresh or whole blood in EDTA, air-dried, stained using the May Grünwald Giemsa method and stored at room temperature before observation under light microscopy to search for intra-erythocytic forms of piroplasms.

Ticks were collected from infested dogs or from the environment by visual picking or flagging. All ticks were stored in 70 % ethanol until identification.

### Ticks identification

Ticks were all identified under light microscopy using morphological identification keys [20, 21]. For ticks of the *R. sanguineus* group, morphological identification was stopped at the group level because of the actual confused taxonomic status of several species inside the group. To unequivocally identify some *R. sanguineus* tick specimens, PCR amplification of the mitochondrial 16S rRNA and 12S rRNA genes (Table 2) and BLAST analysis of the sequences was done as previously described [15]. In particular, all ticks of the *R. sanguineus* group retrieved from the environment were identified by the molecular method because of the greater variety of species possibly collected when using the flagging method (without a selective host trap).

**Table 2:** Primers used in the study

Gene target	PCR target	Name		Fragment length	References
Mitosin gene	*Canis familiaris*	CAN-F	5′-CTTGTCACGGTAAGGTTC-3′	290-bp	[31]
		CAN-R	5′-CTGATGTATTTCCTGCACCAAG-3′		
18S rRNA gene	*Babesia/Theileria spp.*	BTF1 (ext)	5′-GGCTCATTACAACAGTTATAG-3′	930-bp	[22]
		BTR1 (ext)	5′-CCCAAAGACTTTGATTTCTCTC-3′		
		BTF2 (int)	5′-CCGTGCTAATTGTAGGGCTAATAC-3′	800-bp	
		BTR2 (int)	5′-GGACTACGACGGTATCTGATCG-3′		
18S rRNA gene	*Babesia vogeli*	BCV-F	5′-GTGTTCGAGTTTGCCATTCG-3′	422-bp	[15]
		Ba721R	5′-CCCCAGAACCCAAAGACTTTGATTTCTCTCAAG-3′		[32]
Mitochondrial 16S rRNA gene	*Ixodida*	TQ16S + 1 F	5′-CTGCTCAATGATTTTTTAAATTGCTGTGG-3′	320-bp	[33]
		TQ16S-2R	5′-ACGCTGTTATCCCTAGAG-3′		
Mitochondrial 12S rRNA gene	Ticks	Forward	5′-AAACTAGGATTAGATACCCTATTATTTTAG-3′	400-bp	[17]
		Reverse	5′-CTATGTAACGACTTATCTTAATAAAGAGTG-3′		

### DNA extraction from blood and tick samples

DNA was extracted from dog blood samples and a subset of ticks for diagnostic PCR analysis to detect piroplasms. Since *R. sanguineus* and *D. reticulatus* ticks are the only known vectors of piroplasms in Europe to date and the possibility of piroplasm transmission to dogs by other ticks species has never been demonstrated, only ticks belonging to these two species were kept for subsequent analysis. All dog blood samples and all *R. sanguineus* and *D. reticulatus* ticks retrieved from the environment were analyzed. However, for ticks collected from dogs, DNA was extracted from all specimens if the tick infestation level was low and from a subset of specimens selected at random, in numbers proportional to the stage and sex (e.g. larva, nymph, adult male and female) of the ticks found on the host, in cases of heavy infestation. DNA was extracted as previously described [15] and the quality of extracted DNA was assessed by PCR amplification of mitosin or mitochondrial 16S rRNA genes specific for dogs and ticks respectively (Table 2). Quantification of total DNA was systematically performed after each DNA extraction using a spectrophotometer (Nanodrop^®^).

### Molecular methods for piroplasms detection and characterization

All tick and blood DNA samples were screened using two PCR methods : (i) a *Babesia*/*Theileria* genera specific nested PCR method using primer sets BTF1/BTR1 and BTF2/BTR2 that was shown to be very sensitive for the detection of piroplasms on blood [22] and (ii) a specific PCR that was recently validated for detection of *Babesia vogeli* in ticks (Table 2) [15]. The amplification reactions for *Babesia*/*Theileria* genera specific nested PCR were carried out in a thermocycler (Biometra^®^ T gradient, Goettingen, Germany) in 25 μl of reaction mixture containing 30 ng of extracted DNA, 200 μM (each) deoxyribonucleotides, 0.625 U of Hotstartaq DNA polymerase (Qiagen, Hilden, Germany) and 12.5 pmol of each primer in the reaction buffer provided by the manufacturer (Qiagen, Hilden, Germany) [22]. *B. vogeli* specific PCR was performed as explained before [15]. Positive (*B. rossi* and *B. vogeli* DNA) and negative (reaction mix without DNA) controls were used in each PCR assay. Amplified DNA fragments were separated by electrophoresis through 1.5 % agarose gels stained with ethidium bromide and visualized under ultraviolet light. All PCR reactions were strictly performed according to the protocols described in the original papers.

To discriminate between species within the *Babesia*/*Theileria* positive samples detected by nested PCR, a Restriction Fragment Length Polymorphism method (RFLP) was tested on PCR products from the second round of amplification. Amplified fragments of 800-bp 18S rDNA (10 μL) were digested using *Taq*I (65 °C) and *Hinf*I (37 °C) enzymes (Promega, Madison, WI, USA) for 3 h according to a protocol adapted from Carret et al. [23]. Restricted fragments were examined by electrophoresis on 2 % agarose gels and the profiles were compared to those of the 7 species that were detected to date in dog blood samples in Europe *B. caballi*, *B. canis*, *B. gibsoni*, *B. rossi*, *B. vogeli*, *T. equi,* and *T. annae* obtained from infected animals or parasite cultures provided by different laboratories. The identities of these positive controls had been previously confirmed by PCR amplification, sequencing and BLASTN analysis of 18S rRNA gene.

Finally, most *Babesia*/*Theileria* positive PCR products from dogs and ticks were sequenced at BIOFIDAL-DTAMB (FR BioEnvironment and Health, Lyon, France) to assess the possible genetic variability of species and ensure that no other piroplasm with similar restriction profile than the 7 tested was amplified. The sequences were analysed with the BLASTN program (http://blast.ncbi.nlm.nih.gov/Blast.cgi).

### Statistical analysis

Statistical analyses were performed using R software [24]. Prevalences of infection in each area were estimated by calculating the ratio of positive samples divided by the total number of samples analyzed. Prevalences of *B. vogeli* infections in dogs and *R. sanguineus* ticks from Gard, Corsica and Drôme were compared using a Fisher’s exact test with a significance threshold defined as *p* < 0.05.

### Nucleotide sequence accession numbers

Sequences of piroplasms obtained from bloods and/or ticks were deposited in GenBank with the following accession numbers: JX304662 to JX871892 for *B. vogeli*; KC902833 and KC593877 to KC593879 for *B. canis*; and JX454779 for *T. annae*. Mitochondrial 16S rDNA sequences from ticks of the *Rhipicephalus* group affiliated to *R. sanguineus* “temperate” species after BLAST analyses were deposited in GenBank with the following accession numbers: JQ362399 to JQ362409 and JX304684 to JX304708. Mitochondrial 12S rDNA sequences from ticks of the *Rhipicephalus* group affiliated to *R. sanguineus* “temperate” species were deposited in GenBank with accession numbers JX304709 to JX304744. Mitochondrial 16S rDNA sequences from ticks of the *Rhipicephalus* group affiliated to *R. pusillus* after BLAST analyses were deposited in GenBank with accession numbers KC593861 to KC593876. Only sequences of excellent quality were deposited in GenBank.

## Results

### Samples collection and ticks identification

From 2010 to 2012, 155 dogs were selected from which 140 bloods and 635 ticks were retrieved (Tables 1 and 3). In addition, 32 ticks were collected from the environment. Sampling areas involving veterinary clinics were divided in 2 groups: a first group (GA, DR and CO) where samplings were regular and concerned dogs suspected of canine babesiosis as well as healthy dogs infested by ticks and a second group (HE, HG and HP) where samplings were mainly performed in case of canine babesiosis suspicion.

**Table 3:** Results of tick and dog blood collection per study site

Sites code^a^	Sample source	Tick species^b^				Dog blood samples^c^
		*D. reticulatus*	*I. ricinus*	*R. sanguineus* group	Other	
DR	Dogs	0/208 (0.0 %)	18/208 (8.7 %)	190/208 (91.3 %)	0/208 (0.0 %)	25/140 (17.9 %)
HE		NA	NA	NA	NA	9/140 (6.4 %)
HG		30/40 (75.0 %)	4/40 (10.0 %)	6/40 (15.0 %)	0/40 (0.0 %)	15/140 (10.7 %)
HP		3/6 (50.0 %)	2/6 (33.3 %)	0/6 (0.0 %)	1/6 (16.7 %)^d^	6/140 (4.3 %)
GA		0/293 (0.0 %)	0/293 (0.0 %)	293/293 (100.0 %)	0/293 (0.0 %)	49/140 (35.0 %)
CO		0/88 (0.0 %)	3/88 (3.4 %)	85/88 (96.6 %)	0/88 (0.0 %)	36/140 (25.7 %)
Total		33/635 (5.2 %)	27/635 (4.3 %)	574/635 (90.4 %)	1/635 (0.2 %)	
GA2	Environment	0/9 (0.0 %)	0/9 (0.0 %)	9/9 (100.0 %)^e^	0/9 (0.0 %)	
GA5		0/4 (0.0 %)	0/4 (0.0 %)	4/4 (100.0 %)^f^	0/4 (0.0 %)	
GA6		0/19 (0.0 %)	0/19 (0.0 %)	18/19 (94.7 %)^g^	1/19 (5.3 %)^h^	
Total		0/32 (0.0 %)	0/32 (0.0 %)	31/32 (96.9 %)	1/32 (3.1 %)	

^a^Site code (see Table 1 and Fig. 1 for details)

^b^Number of ticks of the corresponding species/Total number of ticks morphologically identified in each area (percentage)

^c^Number of dog blood samples collected in the area/Total number of dog blood samples collected in the study (percentage)

^d^One *Pholeoixodes hexagonus*

^e^Nine *Rhipicephalus sanguineus s.s.* after sequencing of mitochondrial 16S rDNA of ticks

^f^One *Rhipicephalus sanguineus s.s.* and 3 *Rhipicephalus pusillus* after sequencing of mitochondrial 16S rDNA of ticks

^g^Four *Rhipicephalus sanguineus s.s.* and 14 *Rhipicephalus pusillus* after sequencing of mitochondrial 16S rDNA of ticks

^h^One *Haemaphysalis* spp

Ticks were first identified using morphological keys. From the 635 ticks collected from dogs, 574 belonged to the *R. sanguineus* group and 33, 27 and 1 to the species *D. reticulatus*, *Ixodes ricinus* and *Pholeoixodes hexagonus* respectively. From the 32 ticks collected in the environment, 31 were affiliated to the *R. sanguineus* group and 1 to *Haemaphysalis* spp. Among the 574 ticks of the *R. sanguineus* group retrieved from dogs, a subset of 33 ticks (19 from Gard and 14 from Corsica) was selected for molecular identification together with all ticks collected in the environment. After sequencing their mitochondrial 16S rRNA gene, BLASTN analyses confirmed that the 33 specimens retrieved from dogs were affiliated with “temperate” species of *R. sanguineus* having 97 to 100 % identity with *R. sanguineus* specimens from Oklahoma [GenBank: AF081829], Spain [GenBank: Z97884; GenBank: GU553081], Chile [GenBank: GU553077], Uruguay [GenBank: GU553084] and Argentina [GenBank: GU553078]. Among the 31 ticks of the *R. sanguineus* group recovered from the environment, sequence analysis showed that 14 were affiliated with “temperate” species of *R. sanguineus* and 17 with *R. pusillus* (98 to 99 % homology with *R. pusillus* [GenBank: AJ002957 and Z97883]). BLAST analyses of sequences of selected specimens amplified on mitochondrial 12S rRNA gene confirmed previous affiliations.

### Specificity of nested PCR-RFLP for piroplasms detection

**Fig. 2:**
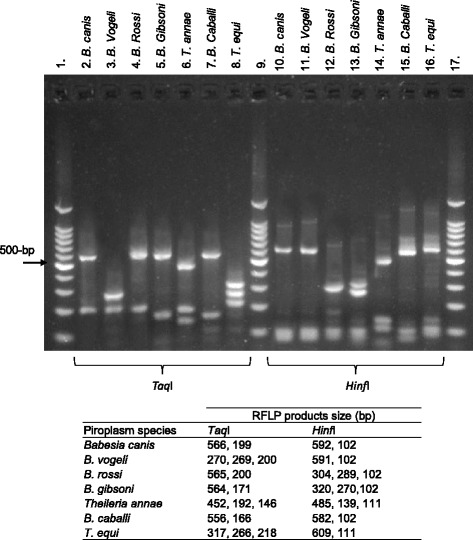
PCR-RFLP profiles of 18S rRNA gene fragments from selected piroplasm species. This method was used to discriminate between *Babesia*/*Theileria* species from ticks or blood samples known to contain piroplasms following nested PCR targeting the 18S rRNA gene. Lanes 2 to 8 and lanes 10 to 16 show PCR-RFLP products for seven piroplasm species known or supposed to infect dogs in France and in Europe digested with endonucleases *TaqI* or *HinfI*, respectively. Lanes 1, 9 and 17 show 100-bp molecular weight markers. Product sizes expected for each piroplasm species after digestion with *TaqI* or *HinfI* enzymes are given in the table

A nested PCR-RFLP method was tested with the seven piroplasm species detected to date in dog blood samples in Europe (Fig. 2). This method confirmed its ability to discriminate the seven *Babesia*/*Theileria* species tested. Its accuracy for detection of *Babesia*/*Theileria* species in dog blood samples as well as in tick samples was established since no inappropriate bands were observed. Consequently, this method was used to screen for piroplasms in dog blood samples and ticks.

### Piroplasms detection in dogs

All blood samples were screened for the presence of piroplasms using *Babesia*/*Theileria* specific nested PCR-RFLP as well as a *B. vogeli* specific PCR method. Results of piroplasm detections in blood samples are shown in Table 4.

**Table 4:** Prevalence of piroplasm infections in dogs

Sample type	Site code^a^	Piroplasm species detected in dog blood samples^b^		
		*B. canis*	*B. vogeli*	Other
Dog blood samples	DR	0/25 (0.0 %)	3/25 (12.0 %)	0/25 (0.0 %)
	HE	6/9 (66.7 %)	2/9 (22.2 %)	0/9 (0.0 %)
	HG	8/15 (53.3 %)	2/15 (13.3 %)	0/15 (0.0 %)
	HP	4/6 (66.7 %)	0/6 (0.0 %)	0/6 (0.0 %)
	GA	0/49 (0.0 %)	12/49 (24.5 %)	1/49 (2.0 %)
	CO	0/36 (0.0 %)	0/36 (0.0 %)	0/36 (0.0 %)
	Total	18/140 (12.9 %)	19/140 (13.6 %)	1/140 (0.7 %)^c^

^a^Site code (see Table 1 and Fig. 1 for details)

^b^Number of positive sample/Number analyzed (percentage) per location

^c^RFLP and sequencing confirmed the affiliation of the protozoa to *Theileria annae*

Among the 140 blood samples, 19 (13.6 %) were positive for *B. vogeli*, 18 (12.9 %) for *B. canis* and 1 (0.7 %) for *T. annae*. No other piroplasm species was detected in blood samples. Twelve of the 19 *B. vogeli* positive dogs were detected with *Babesia*/*Theileria* nested-PCR. In comparison, the pathogen was detected in all positive dogs with *B. vogeli* specific PCR.

In Hérault and Haute-Garonne, both *B. vogeli* and *B. canis* infections occurred in dogs. In Haute-Pyrénées only *B. canis* infections were reported in dogs whereas in Gard and Drôme, *B. vogeli* was the only piroplasm species detected in dogs (except for one case of *Theileria annae* infection in Gard). Finally, in Corsica, none of the 36 dog blood samples analyzed was found to be infected with *Babesia*/*Theileria* species. Overall, the prevalence of *B. vogeli* infection in dogs was higher in Gard (24.5 %) than in Drôme (12.0 %) and Corsica (0.0 %) with a statistical significance (*p* < 0.002).

### Piroplasms detection in *R. sanguineus* and *D. reticulatus* ticks

Results of piroplasms detection in ticks are presented in Table 5.

**Table 5:** Prevalence of piroplasm infections in *R. sanguineus* and *D. reticulatus* ticks

Sample type	Site code^a^	Piroplasm species detected in ticks^b^		
		*B. canis*	*B. vogeli*	Other
*R. sanguineus* ticks	DR	1/56 (1.8 %) - 1M	3/56 (5.4 %) - 1M, 2F	0/56 (0.0 %) - NA
	HG	0/6 (0.0 %) - NA	0/6 (0.0 %) - NA	0/6 (0.0 %) - NA
	GA^d^	3/121 (2.5 %)^c^ - 3F	20/121 ^e^ (16.5 %) - 3M, 16F, 1N	0/121 (0.0 %) - NA
	CO	0/65 (0.0 %) - NA	3/65 (4.6 %) - 1M, 2F	0/65 (0.0 %) - NA
	Total	4/248 (1.6 %) - 1M, 3F	26/248 (10.5 %) - 5M, 20F, 1N	0/248 (0.0 %) - NA
*D. reticulatu*s ticks	HG	2/28 (7.1 %) - 2F	0/28 (0.0 %) - NA	0/28 (0.0 %) - NA
	HP	1/3 (33.3 %) - 1F	0/3 (0.0 %) - NA	0/3 (0.0 %) - NA
	Total	3/31 (9.7 %) - 3F	0/31 (0.0 %) - NA	0/31 (0.0 %) - NA

*NA* not applicable

^a^Site code (see Table 1 and Fig. 1 for details)

^b^Number of positive sample/Number analized (%) - Tick stages of positive samples (M, adult male; F, adult female; N, nymph; NA, not applicable)

^c^The 3 *R. sanguineus* ticks positive for *B. canis* specific PCR-RFLP were also positive for *B. vogeli* species specific PCR

^d^Out of the 121 *R. sanguineus* ticks from Gard selected for piroplasm screening 107 were collected from dogs and 14 from the environment

^e^Among the 20 *B. vogeli* positive ticks, 18 were collected from dogs and 2 from the environment

Among the 588 *R. sanguineus* ticks collected from dogs (*N* = 574) and the environment (*N* = 14 after removal of the ticks finally affiliated to the species *R. pusillus*) 57.3 % were females, 35.2 % were males and 7.5 % were nymphs. Among them, a representative set of 248 *R. sanguineus* ticks were selected for piroplasm screening as follows: 121 from Gard (63.6 % females, 32.2 % males and 4.1 % nymphs; 65 from Corsica (66.2 % females, 27.7 % males and 6.2 % nymphs); 56 from Drôme (66.1 % females, 26.8 % males and 7.1 % nymphs) and 6 from Haute-Garonne. From the 248 *R. sanguineus* specimens screened by PCR, 23 (9.3 %) were positive for *B. vogeli* alone, 1 (0.4 %) for *B. canis* alone and 3 (1.2 %) were doubly-infected with *B. vogeli* and *B. canis*. No other piroplasm species were detected in *R. sanguineus* ticks. Twenty of the 26 ticks infected with *B. vogeli* were females (76.9 %), 5 were males (19.2 %) and 1 was a nymph (3.8 %). From the 26 *B. vogeli* positive ticks, 16 were retrieved from *Babesia*/*Theileria* negative dogs, 5 from *B. vogeli* positive dogs and 3 from dogs whose infection status was not known as their blood was not sampled. The two remaining *B. vogeli* positive ticks were collected in Gard from the environment. All *B. canis* positive *R. sanguineus* ticks were retrieved from *Babesia*/*Theileria* negative dogs. Overall, the prevalence of *B. vogeli* infection in *R. sanguineus* ticks was higher in Gard (10.5 %) than in Drôme (5.4 %) and Corsica (4.6 %) with a statistical significance (*p* < 0.02).

Among the 33 *D. reticulatus* ticks collected from dogs, 31 (93.9 %) were screened by PCR for piroplasms detection and 3 (9.7 %) were positive to *B. canis*. Two of them were retrieved from *B. canis* positive dogs (one from Haute-Garonne and one from Hautes-Pyrénées) and 1 from a *B. vogeli* positive dog from Haute-Garonne. No other piroplasm species were detected in *D. reticulatus* ticks.

### Sequence analysis of *Babesia*/*Theileria* positive samples

BLASTN analysis of 800-bp or 422-bp sequences obtained from *B. vogeli* positive blood and tick samples affiliated these sequences with 99 to 100 % identity to *B. vogeli* from France [GenBank: AY072925], USA [GenBank: AY371198], China [GenBank: HM590440], Venezuela [GenBank: DQ297390], Japan [GenBank: AB083374, AY077719], Romania [GenBank: HQ662635], Egypt [GenBank: AY371197] and Brazil [GenBank: AY371194-AY371196]. Similarly, BLAST analysis of *B. canis* sequences from positive dogs or *R. sanguineus* ticks affiliated these sequences with 99 to 100 % identity to *B. canis* from Croatia [GenBank: AY072926] and Romania [GenBank: HQ662634]. Finally, the sequence obtained from the blood sample which gave an RFLP profile similar to that of *T. annae* showed 99.8 % identity with 18S rRNA gene sequences of *Babesia* sp. ‘Spanish dog’ from Spain [GenBank: AF188001 and AY534602] and USA [GenBank: EU583387], as expected.

### Seasonal distributions of infections

**Fig. 3:**
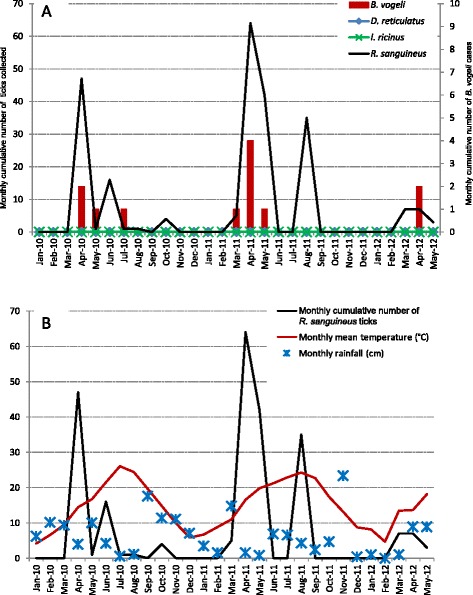
Details of tick collection, *B. vogeli* detection and meteorological records in Gard. Gard is an area recently supposed to be a hot spot of *Babesia vogeli* infection in southern France [15]. **a** Number of *R. sanguineus*, *D. reticulatus* and *Ixodes ricinus* collected each month from dogs and number of canine babesiosis cases caused by *B. vogeli.* **b** Number of *R. sanguineus* collected each month and mean monthly temperature and rainfall. Only ticks retrieved from dogs in veterinary clinics were included in the analysis to avoid potential bias due to the high number of ticks obtained during sampling campaigns in kennels or the environment

As sampling from dogs was done regularly in Gard, it was possible to follow *R. sanguineus* tick activity, through tick infestation in dogs, and canine babesiosis occurrences caused by *B. vogeli* throughout the survey period and relate them to meteorological records (Fig. 3). In 2010 and 2011, *R. sanguineus* ticks were mainly collected from dogs from March to June with a peak in infestation in April. Canine babesiosis cases, mainly caused by *B. vogeli* in this area, were recorded from March to July with a similar peak in observations in April. Interestingly, during the 29 months of the survey, first dog infestations with *R. sanguineus* were detected when the mean monthly temperature went above a threshold value of 10 °C in spring.

All *B. canis* infection cases reported in other areas in the study were recorded in winter or very early spring and when associated with tick infestations, only *D. reticulatus* was found.

## Discussion

Knowledge on epidemiology of canine babesiosis in southern France is scarce, in particular regarding piroplasm species circulating in dogs and tick species involved in their transmission. Moreover, an heterogenic distribution of canine babesiosis cases was previously reported in southern France [12–14] and remain unexplained. Recently we confirmed infection of 4 dogs and 8 *R. sanguineus* ticks with *B. vogeli* in Gard [15], a department from this region, which prompted us to continue and extend the survey to several locations. Thus, a total of 140 dog blood samples and 667 ticks were collected and screened by PCR for piroplasm detection and characterization. The original sampling method used in the study allowed making the link between pathogens detected in dogs and in ticks retrieved from dogs which is essential for accurate results interpretation.

From the 667 ticks collected either on dogs or from the environment in this area, 605 belonged to the *R. sanguineus* group from which 574 were retrieved from dogs confirming the high propensity of this tick species to parasitize dogs in this area. For ticks of the *R. sanguineus* group, because of the controversial taxonomic status of tick species in this group, it was decided to analyze selected specimens using molecular tools. Among the 64 ticks analyzed by sequencing, 47 were finally affiliated to “temperate” species of *R. sanguineus* (= southern lineage) [16–19], in particular all ticks retrieved from dogs, whereas 17 were affiliated to the species *R. pusillus* (all collected from the environment). *R. sanguineus* and *R. pusillus* ticks species obtained by flagging from the environment were all collected along watercourses susceptible to be frequented by dogs. New flagging campaigns should be performed in the future to confirm the possibility of exophilic development of *R. sanguineus* ticks in this area. On the other hand, collection of *R. pusillus* ticks, a species generally known to infest rabbits, may be attributed to the possible presence of such hosts in sampling locations.

Different and complementary methods can be used to evaluate the prevalence of piroplasm infection in dogs. Serology is useless as it lacks species specificity compared to molecular tools that are generally more sensitive and specific [2]. Several PCR-RFLP methods, using *Taq*I and *Hinf*I or other restriction enzymes were previously developed to discriminate between species capable of infecting dogs, notably *B. canis*, *B. gibsoni, B. rossi*, *B. vogeli* and *T. annae* [23, 25, 26]. Molecular methods for piroplasm detection in ticks are less common. Here we modified a very sensitive nested-PCR strategy, previously developed for the detection of *Babesia* and *Theileria* in blood [22] to adapt it to the analysis of tick samples and to allow a specific detection of piroplasms that may infect dogs in Europe. Results clearly proved the accuracy of the modified PCR-RFLP method to detect and differentiate the seven piroplasm species tested both in dog blood and tick samples. The method was then used to screen dog blood and tick samples in combination with a *B. vogeli* specific PCR method that had previously proved its ability to detect the pathogen in ticks [15]. The higher number of *B. vogeli* samples detected by *B. vogeli* specific PCR compared to *Babesia*/*Theileria* nested-PCR showed the higher sensitivity of *B. vogeli* specific method for *B. vogeli* detection in bloods and ticks and support the interest of associating both PCR methods in the study. However, as the sensitivity of the methods was only tested on blood samples the possible impact of the samples (ticks vs blood) on the sensitivity of the methods remains unknown.

Overall, three piroplasm species, *B. vogeli, B. canis*, and *T. annae* were detected in dogs allowing confirmation of the circulation of both *B. vogeli* and *B. canis* in dogs in southern France. For *Theileria annae*, further studies will be needed to assess the prevalence of infection in dogs in this area since only one dog was found infected. No dog was found to be infected with *B. gibsoni* suggesting a low prevalence in France as previously suggested [1, 3]. Both *B. vogeli* and *B. canis* piroplasms were detected in *R. sanguineus* and *D. reticulatus* ticks collected from this area, *B. vogeli* and *B. canis* being the most prevalent species detected in *R. sanguineus* and *D. reticulatus* ticks respectively as expected. Surprisingly, four *R. sanguineus* were positive to *B. canis* infection. To date *D. reticulatus* ticks are the only confirmed vector of *B. canis* [5, 27] and it is assumed that each *Babesia* species is associated with a single tick vector in a delimited geographic area [28]. DNA of *B. canis* was previously found in *R. sanguineus* ticks in Italy and vertical transmission was suggested [29, 30]. The data presented here, in particular, the strong relation between geographical and temporal occurrence in dogs of *B. vogeli* and *R. sanguineus* on one hand and *B. canis* and *D.reticulatus* on the other hand, are highly suggestive of tick-pathogen specificity as generally accepted. However further study should be conducted to assess if *B. canis* infected *R. sanguineus* ticks are capable of transmitting the pathogen to dogs.

An heterogenic distribution of canine babesiosis cases was previously reported in southern France [12–14]. In the study, *B. canis* was the more frequently detected piroplasm species in dogs in Hérault, Haute-Garonne and Hautes-Pyrénées, 3 departments situated in the south-western part of France. In Gard, Drôme and Corsica, all situated in the south-eastern part of France, *B. vogeli* was the main piroplasm species detected in dogs except in Corsica where none of the 36 dog blood samples analyzed was found to be infected with *Babesia*/*Theileria* species. Although the number of blood samples obtained from western Mediterranean basin was limited, these results suggest that *B.canis* and *B. vogeli* could be the main etiological agent of canine babesiosis in south-western and south-eastern France respectively. Moreover, a significantly higher prevalence of *B. vogeli* infection was found in Gard compared to Corsica and Drôme regions, both in dogs (*p* < 0.002) and *R. sanguineus* ticks (*p* < 0.02). Thus, the study provide new data on epidemiology of canine babesiosis in southern France that can partly explain heterogenic distribution of canine babesiosis cases previously reported in this area. However, analysis of a higher number of cases should be conducted in the future to confirm the distribution of pathogens suggested by the study and confront them to biological and ecological characteristics of vectors. Further studies focusing on genetic and microbiota of *R. sanguineus* ticks should be conducted as well to explore other biological interactions that may explain the differences observed.

Six of the sampling locations were situated in the Gard department to enhance knowledge on risk of dogs infection by the protozoan *B. vogeli* that was recently evidenced in this area [15]. In Gard, *B. vogeli* was detected in 12/49 (24,5 %) of dog and in 20/121 (16.5 %) *R. sanguineus* ticks. Among the 20 *B. vogeli* positive ticks, two were non engorged ticks collected by flagging from the environment. These results are consistent with the hypothesized endemicity of *B. vogeli* infections in this area as previously suggested [15]. Interestingly, evidence of a relationship was found between the first infestations of dogs with *R. sanguineus* in spring and an increase in the mean monthly temperature above a threshold value of 10 °C during the 29 months of the study. Such study associating epidemiological and ecological observations should be continued in the future to assess if such a factor could be a useful indicator of *R. sanguineus* infestation and *B. vogeli* infection risk.

## Conclusions

The survey conducted from 2010 to 2012 confirmed that both *B. canis* and *B. vogeli* piroplasms and their respective vectors *D. reticulatus* and *R. sanguineus* parasitize dogs in southern France with differences in distribution probably linked with vectors biotopes. In particular, *B. vogeli* seems to be the most prevalent piroplasm species in the south-eastern part of the country with a higher prevalence of infection found in the Gard area than in Drôme and Corsica. Better understanding of the spatial and temporal distribution of piroplasm species involved in canine babesiosis cases is needed to improve preventive measures and assess the efficacy of treatments and vaccines currently available in France. This implies a better understanding of ecology, genetic and microbiota of vectors to explore other biological interactions that may explain the differences observed.

## References

[1] Irwin PJ (2009). Canine babesiosis: from molecular taxonomy to control. Parasit Vectors.

[2] Irwin PJ (2010). Canine babesiosis. Vet Clin North Am Small Anim Pract.

[3] Solano-Gallego L, Baneth G (2011). Babesiosis in dogs and cats-Expanding parasitological and clinical spectra. Vet Parasitol.

[4] Baneth G, Florin-Christensen M, Cardoso L, Schnittger L (2015). Reclassification of Theileria annae as Babesia vulpes sp. nov. Parasit Vectors.

[5] Beugnet F, Marié JL (2009). Emerging arthropod-borne diseases of companion animals in Europe. Vet Parasitol.

[6] Matijatko V, Torti M, Schetters TP (2012). Canine babesiosis in Europe: how many diseases?. Trends Parasitol.

[7] Beck R, Vojta L, Mrljak V, Marinculic A, Beck A, Zivicnjak T (2009). Diversity of *Babesia* and *Theileria* species in symptomatic and asymptomatic dogs in Croatia. Int J Parasitol.

[8] Fritz D (2010). A PCR study of piroplasms in 166 dogs and 111 horses in France (March 2006 to March 2008). Parasitol Res.

[9] Bourdoiseau G (2006). Canine babesiosis in France. Vet Parasitol.

[10] Martinod S, Gilot B (1991). Epidemiology of canine babesiosis in relation to the activity of *Dermacentor reticulatus* in southern Jura (France). Exp Appl Acarol.

[11] René-Martellet M, Chêne J, Chabanne L, Chalvet-Monfray K, Bourdoiseau G (2013). Clinical signs, seasonal occurrence and causative agents of canine babesiosis in France: Results of a multiregional study. Vet Parasitol.

[12] Lasbleiz M (2007). Situation actuelle de la babésiose canine en France: bilan d’une enquête nationale (in French).

[13] Bourdoiseau G, Renard N (2005). Résultats d’une enquête en France sur les cas suspectés ou confirmés de babésiose chez le chien (in French). Nouveau Prat Vét.

[14] Halos L, Lebert I, Chao I, Vourc’h G, Ducrot C, Abrial D (2013). Questionnaire-based survey on distribution and clinical incidence of canine babesiosis in France. BMC Vet Res.

[15] René M, Chêne J, Beaufils JP, Valiente Moro C, Bourdoiseau G, Mavingui P (2012). First evidence and molecular characterization of *Babesia vogeli* in naturally infected dogs and *Rhipicephalus sanguineus* ticks in southern France. Vet Parasitol.

[16] Dantas-Torres F, Latrofa MS, Annoscia G, Giannelli A, Parisi A, Otranto D (2013). Morphological and genetic diversity of *Rhipicephalus sanguineus sensu lato* from the New and Old Worlds. Parasit Vectors.

[17] Szabó MPJ, Mangold AJ, João CF, Bechara GH, Guglielmone AA (2005). Biological and DNA evidence of two dissimilar populations of the *Rhipicephalus sanguineus* tick group (Acari: Ixodidae) in South America. Vet Parasitol.

[18] Nava S, Mastropaolo M, Venzal JM, Mangold AJ, Guglielmone AA (2012). Mitochondrial DNA analysis of *Rhipicephalus sanguineus sensu lato* (Acari: Ixodidae) in the Southern Cone of South America. Vet Parasitol.

[19] Moraes-Filho J, Marcili A, Nieri-Bastos FA, Richtzenhain LJ, Labruna MB (2011). Genetic analysis of ticks belonging to the *Rhipicephalus sanguineus* group in Latin America. Acta Trop.

[20] Estrada-Peña A, Bouattour A, Camicas JL, Walker AR (2004). Ticks of Domestic Animals in the Mediterranean Region: A Guide to Identification of Species.

[21] Pérez-Eid C (2007). Les Tiques.

[22] Jefferies R, Ryan UM, Irwin PJ (2007). PCR-RFLP for the detection and differentiation of the canine piroplasm species and its use with filter paper-based technologies. Vet Parasitol.

[23] Carret C, Walas F, Carcy B, Grande N, Précigout É, Moubri K (1999). *Babesia canis canis*, *Babesia canis vogeli*, *Babesia canis rossi*: Differentiation of the three subspecies by a restriction fragment length polymorphism analysis on amplified small subunit ribosomal RNA genes. J Euk Microbiol.

[24] R Development Core Team: R (2011). A Language and environment for statistical computing.

[25] Solano-Gallego L, Trotta M, Carli E, Carcy B, Caldin M, Furlanello T (2008). *Babesia canis canis* and *Babesia canis vogeli* clinicopathological findings and DNA detection by means of PCR-RFLP in blood from Italian dogs suspected of tick-borne disease. Vet Parasitol.

[26] Jefferies R, Ryan UM, Muhlnickel CJ, Irwin PJ (2003). Two species of canine *Babesia* in Australia: detection and characterization by PCR. J Parasitol.

[27] Mehlhorn H, Schein E (1984). The piroplasms: life cycle and sexual stages. Adv Parasitol.

[28] Chauvin A, Moreau E, Bonnet S, Plantard O, Malandrin L (2009). *Babesia* and its hosts: adaptation to long-lasting interactions as a way to achieve efficient transmission. Vet Res.

[29] Cassini R, Zanutto S, Frangipane di Regalbono A, Gabrielli S, Calderini P, Moretti A (2009). Canine piroplasmosis in Italy: epidemiological aspects in vertebrate and invertebrate hosts. Vet Parasitol.

[30] Iori A, Gabrielli S, Calderini P, Moretti A, Pietrobelli M, Tampieri MP (2010). Tick reservoirs for piroplasms in central and northern Italy. Vet Parasitol.

[31] Criado-Fornelio A, Martinez-Marcos A, Buling-Saraña A, Barba-Carretero JC (2003). Molecular studies on *Babesia*, *Theileria* and *Hepatozoon* in southern Europe. Part I. Epizootiological aspects. Vet Parasitol.

[32] Kledmanee K, Suwanpakdee S, Krajangwong S, Chatsiriwech J, Suksai P, Suwannachat P (2009). Development of multiplex polymerase chain reaction for detection of *Ehrlichia canis*, *Babesia* spp and *Hepatozoon canis* in canine blood. Southeast Asian J Trop Med Public Health.

[33] Black WC, Piesman J (1994). Phylogeny of hard- and soft-tick taxa (Acari: Ixodida) based on mitochondrial 16S rDNA sequences. Proc Natl Acad Sci U S A.

